# Metabonomics evaluations of age-related changes in the urinary compositions of male Sprague Dawley rats and effects of data normalization methods on statistical and quantitative analysis

**DOI:** 10.1186/1471-2105-8-S7-S3

**Published:** 2007-11-01

**Authors:** Laura K Schnackenberg, Jinchun Sun, Parvaneh Espandiari, Ricky D Holland, Joseph Hanig, Richard D Beger

**Affiliations:** 1 National Center for Toxicological Research (NCTR), Jefferson, AR, 72079, USA; 2 University of Arkansas for Medical Sciences, Little Rock, AR, 72205, USA; 3 Center for Drug Evaluation and Research (CDER), Silver Spring, MD, 20993, USA

## Abstract

**Background:**

Urine from male Sprague-Dawley rats 25, 40, and 80 days old was analyzed by NMR and UPLC/MS. The effects of data normalization procedures on principal component analysis (PCA) and quantitative analysis of NMR-based metabonomics data were investigated. Additionally, the effects of age on the metabolic profiles were examined by both NMR and UPLC/MS analyses.

**Results:**

The data normalization factor was shown to have a great impact on the statistical and quantitative results indicating the need to carefully consider how to best normalize the data within a particular study and when comparing different studies. PCA applied to the data obtained from both NMR and UPLC/MS platforms reveals similar age-related differences. NMR indicated many metabolites associated with the Krebs cycle decrease while citrate and 2-oxoglutarate, also associated with the Krebs cycle, increase in older rats.

**Conclusion:**

This study compared four different normalization methods for the NMR-based metabonomics spectra from an age-related study. It was shown that each method of normalization has a great effect on both the statistical and quantitative analyses. Each normalization method resulted in altered relative positions of significant PCA loadings for each sample spectra but it did not alter which chemical shifts had the highest loadings. The greater the normalization factor was related to age, the greater the separation between age groups was observed in subsequent PCA analyses. The normalization factor that showed the least age dependence was total NMR intensity, which was consistent with UPLC/MS data. Normalization by total intensity attempts to make corrections due to dietary and water intake of the individual animal, which is especially useful in metabonomics evaluations of urine. Additionally, metabonomics evaluations of age-related effects showed decreased concentrations of many Krebs cycle intermediates along with increased levels of oxidized antioxidants in urine of older rats, which is consistent with current theories on aging and its association with diminishing mitochondrial function and increasing levels of reactive oxygen species. Analysis of urine by both NMR and UPLC/MS provides a comprehensive and complementary means of examining metabolic events in aging rats.

## Background

Metabonomics has been widely used to investigate new biomarkers of disease at different stages and drug-induced toxicities using analytical techniques including NMR [1-3] and HPLC/MS [4-7]. However, it is important to note that the metabolite profile in animal urine can be affected by various environmental factors that include gender, age, species, diet, water intake, and gut microflora [8-10]. Age can affect a metabolic profile due to the non-linear development of various organs, which also affects the level of biliary activity. The aforementioned environmental factors will also influence the metabolic profile and impact statistical and quantitative analysis of the data. As a result, choosing an inappropriate control population and data normalization factors for a metabonomics study may reveal biased biomarkers of disease or drug toxicity. It is recognized that some preprocessing of metabonomics data is required, however, the question remains as to the best method for normalizing the data prior to statistical analyses [11,12]. The importance of carefully selecting age-matched controls has been previously noted [13-15]. As such, it is necessary to first study the age-related changes in the urinary metabolites of control animals in order to facilitate drug toxicity evaluation using metabonomics techniques.

To date, the bulk of age-related studies (associated with animal gender, strain and diurnal effects) have been performed using technologies including both high field NMR and LC/MS combined with advanced chemometric and pattern recognition techniques [5,13,15-18]. Age-related changes in urinary metabolites have been observed over a five month period using normal Wister-derived rats as the model [15]. In addition, the same group also investigated the differences in metabolite profiles between male Zucker (fa/fa) obese and Wister-derived rats at 12 weeks old and over 4 to 20 weeks of age [15,18].

The present study was originally designed for tracking early biomarkers of pediatric drug toxicity at each major development stage using male Sprague-Dawley (SD) rats at 25, 40 and 80 days old as models of human toddlers, young and mature adults, respectively. Here, we evaluate the effects of different normalization procedures on the statistical and quantitative analysis of NMR data within a group of control, male SD rats at three ages. We also describe the NMR and UPLC/MS-observed changes in the endogenous metabolic profile associated with aging and development in the same groups of non-treated male rats. The results reported here are a by-product of ongoing studies conducted at the Center for Drug Evaluation and Research (CDER).

## Results

### Effects of normalization factor on PCA of NMR data

**Figure 1:**
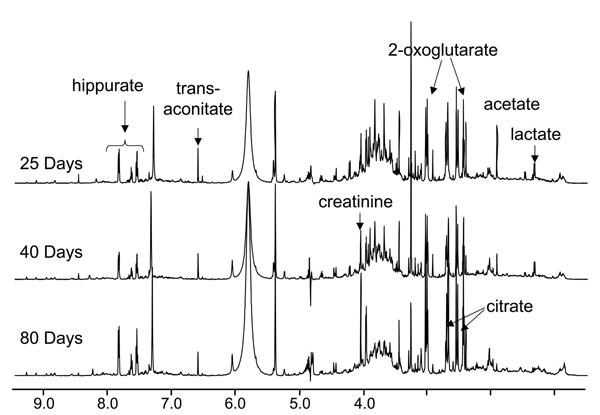
**Representative NMR spectra of urine**. Overlaid representative NMR spectra of predose 0 hour urine from 25, 40 and 80-day old SD rats.

**Figure 2:**
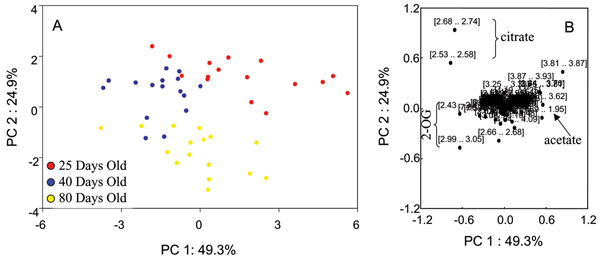
**PCA scores and loadings normalized to total intensity**. (A) 2D PCA scores and loadings plots of NMR data normalized to total spectral intensity from urine samples of 25-day (red circles), 40-day (blue circles), and 80-day (yellow circles) old control SD rats at all timepoints. (B) The PC1 and PC2 loadings for the negative NMR PCA analysis.

**Figure 3:**
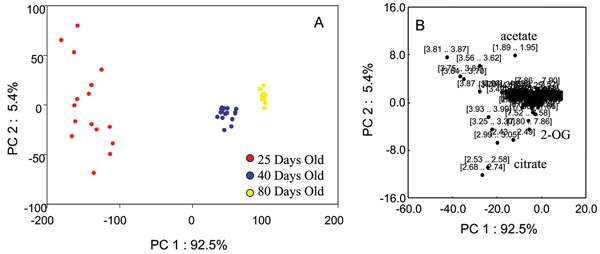
**PCA scores and loadings normalized to weight**. (A) 2D PCA scores and loadings plots of NMR data renormalized by animal weight from urine samples of 25-day (red circles), 40-day (blue circles), and 80-day (yellow circles) old control SD rats at all timepoints. (B) The PC1 and PC2 loadings for the negative NMR PCA analysis.

**Figure 4:**
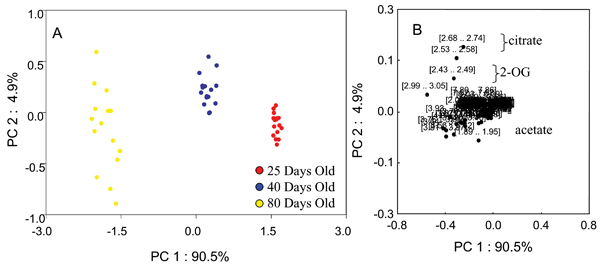
**PCA scores and loadings normalized to 1/weight**. (A) 2D PCA scores and loadings plots of NMR data renormalized by 1/animal weight from urine samples of 25-day (red circles), 40-day (blue circles), and 80-day (yellow circles) old control SD rats at all timepoints. (B) The PC1 and PC2 loadings for the negative NMR PCA analysis.

**Figure 5:**
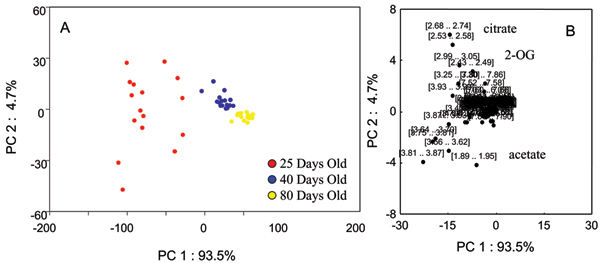
**PCA scores and loadings normalized to [Creatinine]**. (A) 2D PCA scores and loadings plots of NMR data renormalized by the concentration of creatinine in the individual samples from urine samples of 25-day (red circles), 40-day (blue circles), and 80-day (yellow circles) old control SD rats at all timepoints. (B) The PC1 and PC2 loadings for the negative NMR PCA analysis.

Figure 1 shows representative NMR spectra of predose (0 hour) urine from rats aged 25 days, 40 days, and 80 days with many of the age-related metabolites annotated. Additional File 1 shows the 3D PCA plot analysis of the 0 hr urine spectra from rats within the three different age groups. A clear separation of these three age groups with the 40 days old group located between the 25 days old and 80 days old groups is seen. The PCA plot presented in Additional File 1 was generated from integral bins for each spectrum normalized to the total spectral intensity of the individual spectra. The effects of other normalization factors including age and creatinine (Crea) concentration on the statistical analyses were also investigated. Figures 2, 3, 4, 5 show the effects of data normalization procedures on the statistical analysis of the NMR metabonomics dataset. Figure 2 shows the PCA scores and associated loadings plot for the initial table of integrals generated in ACD/Labs 1D NMR Manager (ACD/Labs, Toronto, CA) and normalized by total spectral intensity. There is a clear separation in the data based upon age with the 25 and 40 day old groups separating along principle component (PC) 1 and the 80 day old group separating from the 25 and 40 day old groups along PC2. The associated loadings plot shows the observed clustering is primarily due to changes in 2-oxoglutarate, citrate, and acetate. Figures 3 and 4 show the effects of normalizing by animal weight and 1/weight has on PCA. There is a clear separation of the three age groups along PC1 as the use of weight as a normalization factor amplifies many age-related differences. Finally, Figure 5 shows the effect of normalizing by the concentration of creatinine has on PCA analysis. The 25 and 40 day old groups form tight clusters while the 80 day old group shows a bit more scatter. The three age groups are separated along PC1 based primarily on the differences in creatinine concentration, which changes throughout the lifespan reflecting again the age-related changes between the three age groups.

### Effects of normalization factor on quantitative analysis of NMR data

The concentrations obtained by analysis with Chenomx NMR Suite (Chenomx, Calgary, Canada) are determined relative to the sodium 3-trimethyl-silyl-[2,2,3,3,-d_4_]propionate (TMSP) concentration within each sample. The TMSP concentration is a value that is defined by the user prior to analysis of the spectrum. However, the effects of TMSP volatility and other factors that may result in variation of the TMSP concentration are not accounted for in determining the concentrations of the metabolites. Select metabolites from urine samples obtained from 80 day old control SD rats in two separate studies were quantified and compared. The concentrations were additionally normalized either by the creatinine concentration in the individual samples or by the TMSP peak intensity/total spectra intensity and the datasets compared.

Table 1 reports the concentrations for select metabolites from normalization either by the creatinine concentration in the individual samples or by the TMSP peak intensity/total spectra intensity for the 80 day old age group. Similar results were obtained for the other two age groups. The first two columns report the concentrations in units of micromolar (μM) as determined from Chenomx analysis based solely on the TMSP concentration that was defined by the user. The second two columns report the concentrations normalized to the internal creatinine concentration (μM/[Crea] (μM)). Finally, the last two columns report the concentrations normalized to TMSP peak intensity/total spectral intensity in units of μM. This normalization method takes into account any variation in the TMSP peak concentration between samples and between studies and provided the most consistent results between studies.

**Table 1:** Normalization effects on metabolite concentration data. Effects of normalization on the quantitation of metabolite concentrations. Concentrations are reported in units of μM in columns 2, 3, 6, and 7. The concentration units in columns 4 and 5 are reported as μM/[Creatinine] (μM).

Postulated identity	Study 1 (μM)	Study 2 (μM)	Study 1 Normalized to Creatinine (μM)/[Crea] (μM)	Study 2 Normalized to Creatinine (μM)/[Crea] (μM)	Study 1 Normalized to TMSP/total intensity (μM)	Study 2 Normalized to TMSP/total intensity (μM)
2-OG	3915 ± 2459	6261 ± 2524	1.24 ± 0.04	1.48 ± 0.55	62.5 ± 24.8	82.3 ± 25.9
Acetate	986 ± 1145	440 ± 547	0.33 ± 0.36	0.19 ± 0.24	16.9 ± 20.6	11.1 ± 13.7
Citrate	6563 ± 3896	6436 ± 2182	2.10 ± 0.58	1.61 ± 0.50	104.6 ± 36.5	90.3 ± 24.6
Creatine	329 ± 544	550 ± 811	0.12 ± 0.20	0.27 ± 0.25	7.1 ± 12.3	15.9 ± 14.8
Creatinine	3029 ± 1161	3710 ± 772	N/A	N/A	50.9 ± 13.5	57.0 ± 3.0
Formate	342 ± 167	403 ± 311	0.11 ± 0.04	0.10 ± 0.06	5.7 ± 2.3	5.4 ± 3.0
Hippurate	2209 ± 1118	3489 ± 1046	0.74 ± 0.26	0.81 ± 0.23	37.2 ± 15.9	45.5 ± 12.1
Oxalacetate	766 ± 298	514 ± 180	0.28 ± 0.12	0.15 ± 0.07	13.5 ± 5.7	8.7 ± 3.6
Taurine	1011 ± 798	3187 ± 1611	0.35 ± 0.29	0.83 ± 0.47	19.5 ± 19.3	46.9 ± 25.2
trans-Aconitate	1329 ± 528	1582 ± 425	0.44 ± 0.06	0.38 ± 0.11	22.3 ± 6.1	21.5 ± 60.0

### NMR analysis of urine

Total intensity normalization was utilized for data analysis in this metabonomics study due to the minimized bias between the three age groups. As mentioned above, Additional File 1 shows the results of PCA analysis of the NMR-based metabonomics data from the three age groups. This observation was similar to results obtained by UPLC/MS (data shown later). The loadings plot (data not shown) represents the chemical shifts for molecules responsible for the significant contributions to the age clustering. In this study, citrate, 2-oxoglutarate, and acetate were molecules associated with aging and development of the animals.

Table 2 shows a list of normalized metabolite concentrations (μM) that were identified by Chenomx and showed an age-dependence in urine of the SD rats in this study. As shown in the PCA loadings plot, 2-oxoglutarate and citrate had the largest contributions to the age-group separations with these metabolites increasing in concentration as the animals aged. Some Krebs cycle intermediates were decreased during rat aging and development. These molecules included acetate, fumarate, oxalacetate, pyruvate, and trans-aconitate through its conversion to cis-aconitate, all of which had lower normalized concentrations in the urine of 80 days old rats when compared to concentrations observed in 25 days old rats. Other metabolites related to the Krebs cycle including glutamate and glutamine (related to 2-oxoglutarate) and formate (related to pyruvate) had lower normalized concentrations in urine from the 80 days old rats.

**Table 2:** NMR detected metabolic biomarkers of age. Age-related metabolite normalized concentrations reported as μM detected by NMR in urine.

Postulated identity	25 Days (μM)	40 Days (μM)	80 Days (μM)
2-Oxoglutarate	48.3 ± 16.6	85.3 ± 14.3† p = 2.0E-07 vs. 25	62.5 ± 24.8‡ p = 4.0E-03 vs. 40
Acetate	24.1 ± 26.7	9.1 ± 5.4† p = 4.3E-02 vs. 25	16.9 ± 20.6
Alanine	5.0 ± 1.9	2.7 ± 0.6† p = 1.9E-04 vs. 25	3.0 ± 2.1† p = 7.6E-03 vs. 25
Betaine	8.2 ± 3.5	9.2 ± 5.6	2.5 ± 1.1†‡ p = 9.1E-06 vs. 25 p = 2.4E-04 vs. 40
Citrate	94.6 ± 48.8	164.2 ± 33.7† p = 7.0E-05 vs. 25	104.6 ± 36.5‡ p = 4.1E-05 vs. 40
Creatine	11.7 ± 2.5	4.2 ± 2.3† p = 1.0E-09 vs. 25	7.1 ± 12.3
Creatinine	18.4 ± 3.5	26.3 ± 4.0† p = 1.6E-06 vs. 25	50.9 ± 13.5†‡ p = 4.5E-08 vs. 25 p = 1.6E-06 vs. 40
Formate	11.7 ± 7.4	7.3 ± 1.7† p = 3.2E-02 vs. 25	5.7 ± 2.3†‡ p = 6.4E-03 vs. 25 p = 4.0E-02 vs. 40
Fumarate	1.9 ± 0.9	1.6 ± 0.3	1.0 ± 0.4†‡ p = 1.0E-03 vs. 25 p = 1.6E-05 vs. 40
Glutamate	2.5 ± 1.2	1.7 ± 0.3† p = 1.0E-02 vs. 25	1.2 ± 0.3†‡ p = 2.9E-04 vs. 25 p = 5.7E-05 vs. 40
Glutamine	3.7 ± 1.2	2.4 ± 0.5† p = 5.4E-04 vs. 25	2.6 ± 0.8† p = 2.8E-03 vs. 25
Hippurate	25.4 ± 13.9	40.6 ± 6.4† p = 6.9E-04 vs. 25	37.2 ± 15.9† p = 3.3E-02 vs. 25
Oxalacetate	36.1 ± 15.5	20.0 ± 5.7† p = 9.4E-04 vs. 25	13.5 ± 5.7†‡ p = 2.7E-05 vs.25 p = 2.9E-03 vs. 40
Pyruvate	2.2 ± 2.1	1.4 ± 0.5	1.1 ± 0.7
Succinate	8.6 ± 2.1	6.9 ± 1.2† p = 6.5E-03 vs. 25	8.3 ± 2.9
Taurine	2.7 ± 2.1	1.6 ± 0.7	19.5 ± 19.3†‡ p = 3.4E-03 vs. 25 p = 2.1E-03 vs. 40
trans-Aconitate	26.2 ± 4.9	30.2 ± 4.4† p = 2.1E-02 vs. 25	22.3 ± 6.1‡ p = 2.8E-04 vs. 25

### UPLC/MS in positive mode

**Figure 6:**
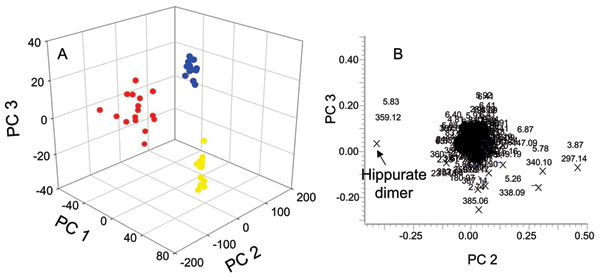
**Positive mode UPLC/MS PCA scores and loadings plots**. (A) Three dimensional PCA trajectory plots for UPLC/MS data in the positive ionization mode data from 25-day (red circles), 40-day (blue circles), and 80-day (yellow circles) old SD rats at all time points. (B) The PC2 and PC3 loadings for the positive UPLC/MS PCA analysis.

Typical total ion chromatograms (TIC) resulting from positive mode analysis of urine from a 25, 40, and 80 day old rat are displayed in Additional File 2. The mass spectrum of hippuric acid (*m/z *180.0661, with retention time (t_R_) 5.85 min, mass accuracy 0.7 ppm) is displayed as an inset in Additional File 2. From simple visual examination of these chromatograms, some age-related alterations to the urinary profiles were readily observed with decreased unknown ion *m/z *349.1858 at t_R _5.28 min and increased hippuric acid *m/z *180.0661 at t_R _5.85 min associated with aging. PCA analysis was further applied in order to fully characterize the metabolic changes and is shown in the 3D PCA scores plot (Figure 6A) and associated loadings plot (Figure 6B). A clear clustering of the data according to age was observed with 40 days old rats located in the middle of the 25 days old and 80 days old groups consistent with the PCA results obtained by NMR analysis. The dimer of hippuric acid (*m/z *359.12 at t_R _5.85 min) had the highest contribution to the age-based clustering based on its PCA significance and is shown in the loadings plot (Figure 6B).

Other positive ions that contributed most to the age-related clustering according to their PCA significance are shown in Table 3 and Table 4. These ions were visually re-examined through extracted ion chromatograms (EIC) for each *m/z *value in order to exclude false signals that may have been selected by the software. Hippuric acid (*m/z *180.0661 at t_R _5.83 min) and kynurenic acid salt (*m/z *212.0324 at t_R _2.14 min) were detected as ions that increased in older rats. Their identification was based on the detected exact mass accuracy and further confirmed with authentic standards and retention times. The ion with *m/z *231.1695 at t_R _2.25 min was also detected as an up-regulated compound. Table 3 shows other ions that were detected as being down-regulated biomolecules, which included those with retention times at 3.87, 3.88, 4.91, 5.24, 5.91, 6.82, 6.87 and 8.91 min and corresponding *m/z *297.1445, 303.0738, 305.1582, 349.1832, 431.0992, 233.0963., 447.0938, and 410.1856, respectively.

**Table 3:** Principal positive ions that increase during aging. Retention time, measured *m/z*, calculated *m/z*, postulated identity, and elemental composition of positive ions that increase during aging.

Retention time (min)	Measured *m/z *(Da)	Calculated *m/z *(Da)	Postulated identity (with t_R _of standard)	Elemental composition of [M+H] ion
2.14	212.1061	212.0324	Kynurenic acid Na	C_10_H_7_NO_3_Na
2.25	231.1701	231.1695		C_9_H_21_N_5_O_2_
5.83	180.0659	180.0661	Hippuric acid (5.83)	C_9_H_10_NO_3_

**Table 4:** Principal positive ions that decrease during aging. Retention time, measured *m/z*, calculated *m/z*, postulated identity, and elemental composition of positive ions that decrease during aging.

Retention time (min)	Measured *m/z *(Da)	Calculated *m/z *(Da)	Postulated identity (with t_R _of standard)	Elemental composition of [M+H] ion
3.87	297.1445	297.1450		C_14_H_21_N_2_O_5_
3.88	303.0738			
4.91	305.1582	305.1576		C_12_H_26_O_7_Na
5.24	349.1832	349.1838		C_14_H_30_O_8_Na
5.91	431.0992			
6.82	233.0963			
6.87	447.0938			
8.91	410.1856			

### UPLC/MS in negative mode

**Figure 7:**
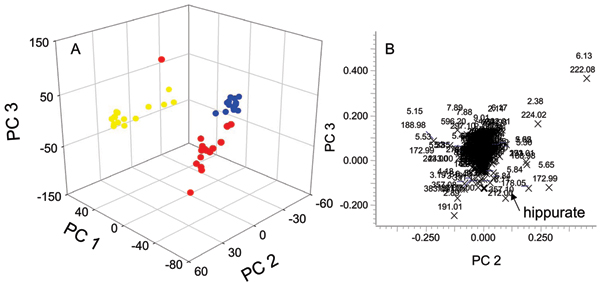
**Negative mode UPLC/MS PCA scores and loadings plots**. (A) Three dimensional PCA scores plot obtained from UPLC/MS data acquired from SD-rat urine samples collected from data from 25-day (red circles), 40-day (blue circles), and 80-day (yellow circles) old SD rats at all time points in the negative ionization mode. (B) The PC2 and PC3 loadings for the negative UPLC/MS PCA analysis.

Typical total ion chromatograms resulting from negative mode analysis of urine from a 25, 40 and 80 day old rats are displayed in Additional File 3. The mass spectrum of hippuric acid (*m/z *178.0500, t_R _5.85 min, mass accuracy 2.8 ppm) is shown as an inset in Additional File 3. From visual examination of the chromatograms in Additional File 3, some age-related differences to the urinary profiles *e.g. *ion with *m/z *222.0764 at t_R _6.14 were readily observed as increased with aging. PCA analysis was also employed to fully characterize the metabolic changes related to animal aging and the developmental process. The PCA 3D scores plot and 2D loadings plot are shown in Figure 7. Similar to the results from positive mode LC/MS, a clear clustering of data according to age was observed with 40 days old rats located between the 25 days old and 80 days old groups as noted in Figure 7A. The ion with *m/z *222.0764 at 6.14 min had the highest contribution to the clustering based on the PCA loadings as noted in the loadings plot shown in Figure 7B.

All major ions with large contributions to the separation along with their potential atomic compositions and identities are listed in Table 5. Similar to positive ion analysis, these ions were manually inspected from the raw data through the extracted ion chromatogram in order to assure the 'true' ion signal was detected and the detected mass accuracy. Suberic acid (*m/z *173.0800 at t_R _6.93, mass accuracy 8.1 ppm) was identified as increased with age based on the accurate mass detection and verification by an authentic standard. Ferulic acid sulfate (*m/z *273.0060 at t_R _5.50, mass accuracy 3.3 ppm), an important metabolite of ferulic acid, was detected as increased. An ion with m/z 247.1055 at t_R _4.62 was identified as 6-hydroxymelatonin by comparison with a standard. Other unidentified ions detected as increased with aging included ions with t_R_'s at 2.38, 2.40, 3.53, 4.78, 4.85, 5.50 and 5.53 min and corresponding *m/z *224.0210, 468.0941, 252.0544, 276.0547, 308.1167 and 275.0220, respectively. Identification of these unidentified ions is still ongoing. One negative ion peak at t_R _1.87 min and *m/z *387.115 showed a tendency to decrease over age. A peak at 3.19 min and *m/z *191.0147, identified as citric acid was found to be at a maximum in 40 days old SD rats; an observation that was also seen by NMR.

**Table 5:** Principal negative ions that increase during aging. Retention time, measured *m/z*, calculated *m/z*, postulated identity, and elemental composition of negative ions that increase during aging.

Retention time (min)	Measured *m/z *(Da)	Calculated *m/z *(Da)	Postulated identity (with t_R _of standard)	Elemental composition of [M+H] ion
2.38	224.0210			
2.40	468.0941			
3.53	252.0544			
4.62	247.1055	247.1083	6-Hydroxymelatonin (4.59)	C_13_H_15_N_2_O_3_
4.78	276.0547			
4.85	308.1167			
5.50	273.0063	273.0069	Ferulic acid sulfate	C_10_H_9_SO_7_
5.53	275.0220	275.0225		C_10_H_11_SO_7_
6.14	222.0764			
6.93	173.0806	173.0814	Suberic acid (6.92)	C_8_H_13_O_4_

## Discussion

The urine output, which will vary between animals, will affect the total intensity of each spectrum making it necessary to apply a normalization step to account for any variation prior to statistical analysis. Normalization by total intensity of the NMR spectra is generally the preferred method [16,19-23]. Normalization by total intensity is used to compensate for different dilution factors when analyzing urine samples. Another common method is to normalize by the internal concentration of creatinine. The creatinine concentration is assumed to be a constant value under conditions where the kidney is functioning normally and normalization by creatinine is intended to account for urine dilution effects. The practice of normalizing clinical parameters by creatinine is still widely used in kidney diagnostics. However, there has been some question as to the validity of correcting by creatinine concentration and specifically whether normalization by creatinine introduces an age-related bias into clinically measured parameters [24]. Since the amount of food and water consumed by rats is directly correlated and preclinical dosing is often normalized to weight, we evaluated normalization by the weight and by 1/weight for each rat.

In either case where weight was used as a normalization factor, the primary separation in PCA was along PC1 and due primarily to the differences in animal weight for the three age groups. The average weight for the 80 day old group was 430% greater than the average weight for the 25 day old group. Clearly, normalization by weight introduces a bias that masks other biologically relevant processes. However, if the effects of age alone are of interest, normalizing by animal weight will clearly amplify the age-related effects. The average creatinine concentration also varied with age with a 47% increase between the 25 and 80 days old groups. Creatinine is a breakdown product of creatine, which is a major component of muscle. Studies have shown that the urinary creatinine concentration increases in humans as they age and then declines later in life [25,26]. The creatinine concentration is closely correlated with muscle mass and nutritional status. In this case, normalizing the integral data by creatinine is again amplifying the effects of age over any other underlying biological processes.

The investigation of normalization factor indicated that the greater the correlation between normalization factor and age, the greater the separation between the age groups in PCA analyses. Correlations between age and the normalization factor were determined for comparison. The normalization factor that showed the least age dependence was total NMR intensity with a correlation between age and PC1 of -0.25. The corresponding PCA plots based on total intensity normalization also showed the smallest separation between age groups although the three groups were still clearly separated. The PCA based on integral bins normalized to creatinine levels had a correlation between age and PC1 of 0.81. The PCA of spectra normalized to animal weight had a correlation between age and PC1 of 0.83 while normalization by 1/weight had a correlation between age and PC1 of -0.99. In effect, normalization by creatinine concentration changes each individual spectrum by that factor. For example, a spectral bin that had a high correlation with creatinine concentration before normalization would lose that age-related dependence after normalization. Regions of the urine spectra which were unrelated to aging after normalization to total intensity would now have an age-related dependence equivalent to the creatinine age dependence after normalization of the spectra to creatinine. The same principles apply for normalization by to weight and 1/weight. These results indicate that the greater the variation and the higher the correlation of the normalization factor with age, the greater the bias that is introduced into further statistical analysis. Since total intensity shows the least change between the three age groups, the bias is minimized. Thus, total intensity normalization was utilized for data analysis in this metabonomics study. Indeed, result from PCA analysis of NMR-based data was consistent with that of LC/MS based data.

In comparing the effects of different compounds from unique studies, it is important to carefully consider how to quantitatively normalize the data. Select metabolites from the 0 hr urine spectra of 80 day old rats in two distinct datasets were quantified and compared. Prior to any normalization procedure, obvious variability in the concentrations between the two datasets was noted. The concentrations were expected to be quite similar based upon the fact that the animals used in both studies were the same strain and housed and fed in the same manner. As known, the concentrations obtained by analysis with Chenomx NMR Suite (Chenomx, Calgary, Canada) are determined relative to the sodium 3-trimethyl-silyl-[2,2,3,3,-d_4_]propionate (TMSP) concentration within each sample. The TMSP concentration is a value that is defined by the user prior to analysis of the spectrum. However, the effects of TMSP volatility and other factors that may result in variation of the TMSP concentration are not accounted for in determining the concentrations of the metabolites. For example, it was determined that the TMSP concentration differed by a factor of almost two between the two studies, which was not accounted for in the analysis.

Initially, the creatinine concentration was used for normalization. However, the creatinine concentration is also reported relative to the TMSP concentration, which was noted above to differ between the studies. Further, the creatinine concentration increases slightly with age, which will affect any comparisons between the datasets. Concentrations were then normalized by TMSP peak intensity/total spectral intensity. This normalization method takes into account any variation in the TMSP peak concentration between samples and between studies and provided the most consistent results although some variations still exist. The variations noted between the two data sets normalized to TMSP peak intensity/total spectral intensity may be related to other environmental factors like diet. While the two studies were run in the same manner, the samples were collected almost a year apart so the composition of the food could have differed slightly. Further, the animals were provided access to water and chow *ad libitum *so there was no dietary control. Regardless, the results indicate that normalization by total spectral intensity and TMSP intensity is more realistic and scientifically-based.

In addition to addressing factors that affect statistical and quantitative analysis, NMR and MS analyses were used to investigate age-related changes in the metabolic profiles at ages 25, 40, and 80 days. Result from PCA analysis of NMR data based on total intensity normalization was similar with that from LC/MS data (Additional File 1, Figures 6 and 7). These observations confirmed that NMR data based on total intensity normalization truly reflected the effects of age over any other underlying biological processes.

Comprehensive and complementary metabolic information in aging rats can be obtained with the combination of NMR and UPLC/MS, since they possess different principal techniques. NMR data demonstrated that many metabolites associated with the Krebs cycle decreased with age while citrate and 2-oxoglutarate, also associated with the Krebs cycle, were most elevated in the 40 day old animals. Nonetheless, the fact that many metabolites in the Krebs cycle decreased while a few increased as the rats aged cannot be fully explained by simple dietary or activity changes. Normalized levels of creatine were also decreased with age. Normalized levels of hippurate, creatinine, and taurine increased when going from 25 days to 80 days old similar to the results presented by Williams and coworkers [15]. Hippurate is related to the gut microflora level with the increased concentration in the older rats indicating that the gut microflora develops as a rat ages. This trend in hippurate levels has been previously noted [15]. Age-related increases in taurine levels were also previously observed in a study of Wistar rats [15]. Taurine has been reported as a marker of liver function and the increase in taurine may be related to a decrease in liver function [27-29]. Further, taurine has been proposed to provide some protection to tissue against reactive oxygen species (ROS) [30]. Serum creatinine is a known marker of renal function that increases in concentration in the urine as renal function decreases [31,32]. The fact that creatinine concentration changes during rat aging makes normalization based on this molecule unsuitable for detecting age related biomarkers.

UPLC/MS data collected in positive mode showed that the dimer of hippuric acid had the highest contribution to the age-based clustering based on its PCA significance. This result was consistent with the observation in other age-related metabonomics studies [15]. The hippuric acid trend plot (data not shown) shows a lower concentration for the 25 days old group relative to the older groups (40 days old and 80 days old), which had similar concentrations. This is most likely caused by the relative lower total gut microflora content in younger animals. Hippuric acid and the ion at *m/z *231.1695 were also detected as age-related compounds present in the urine in the study by Williams *et al. *[15]. Differences in the other ions reported here from those detected by Williams and coworkers [15] were most likely due to the fact that different strains of animals (Wistar-derived vs. SD rats) and different ages (4 to 20 weeks Wistar rats versus 25 day, 40 day, and 80 day SD rats) were used in the individual studies. As such, hippuric acid and the ion at *m/z *231.1695 could be postulated as age-related biomarkers for all strains of rats. Suberic acid and ferulic acid sulfate, an important metabolite of ferulic acid, were detected as increased in negative mode UPLC/MS spectra. The elevation in ferulic acid may be the result in response to an increased need for antioxidants due to higher levels of reactive oxygen species (ROS) that are present as the animal ages. Ferulic acid may offer significant health benefits through its antioxidant and anti-cancer activity [33,34]. The ion identified as 6-hydroxymelatonin also increased with age. The presence of higher concentrations of suberic acid in older rats may reveal increased fatty acid oxidation [36,37], while 6-hydroxymelatonin would support increased antioxidant activity of melatonin. This increase is likely related to increased antioxidant activity of melatonin throughout the aging process.

## Conclusion

This study compared different normalization methods for the NMR-based metabonomics data. It was shown that the method of normalization has a great effect on both the statistical and quantitative analyses. The age-dependent clustering was dependent on the normalization procedure used prior to PCA of the NMR integral data. The normalization of NMR data altered the relative position of significant PCA loadings but did not alter which chemical shifts and associated metabolites had the highest loadings. The greater the normalization factor was related to age, the greater the separation between age groups was observed in subsequent PCA analyses. The normalization factor that showed the least age dependence was total NMR intensity. These results affirm that it is necessary to consider the age of the animal and the best way to normalize data when comparing results from different metabonomics studies.

In addition to investigating the effects of normalization on NMR-based metabonomics data, the age dependence on the composition of metabolites was assessed using both NMR and UPLC/MS methods. PCA analyses indicated very little time dependence on the NMR and UPLC/MS spectra of urine obtained from control SD rats administered saline daily for three days and from pre-dose (0 hours) animals. However, a significant age-related effect was observed. Decreased levels of many Kreb's cycle intermediates along with increased levels of oxidized lipids and antioxidants in older SD rats is consistent with current theories about how ROS and mitochondrial metabolism play a major role in aging [22,35]. The noted increase of suberic acid, 6-hydroxymelatonin, and ferulic acid sulfate along with NMR detected taurine related to aging could represent a protective mechanism as reactive oxygen species also increase.

## Methods

### Sample collection

Male Sprague-Dawley (SD) rats (Harlan, Indianapolis, IN) of ages 25, 40, and 80 days old were used for the study. The animals were maintained in plastic metabolism cages in a controlled environment at 22°C with a 12-h light-dark cycle. Rats were fed Purina rodent laboratory chow (Purina Mills, St. Louis, MO) and provided water *ad libitum*. Urine was collected at 0, 24, 48, and 72 hours from control SD rats that were administered saline daily. Predose (0 hours) urine was also collected and analyzed from rats used in a toxicity study.

### NMR data acquisition

Samples were prepared by combining 400 μL of urine, 200 μL of sodium phosphate buffer (pH = 7.4) and 60 μL of a mixture containing 10 mM TMSP (sodium 3-trimethyl-silyl-[2,2,3,3,-d_4_]propionate) and 100 mM imidazole. Proton (^1^H) NMR spectra were acquired on a Bruker Avance spectrometer operating at 600.133 MHz for proton and equipped with a triple resonance cryoprobe. Water suppression was achieved through application of the Bruker "noesypresat" pulse sequence, which irradiates the water resonance during a delay time (d1 = 2 sec) and a mixing time (d8 = 100 msec). For each sample, 32 scans were collected into 65,536 data points. A spectral width of 9615.39 Hz was utilized with an acquisition time of 3.41 seconds.

### Liquid chromatography

Aliquots of 100 μL of rat urine were centrifuged at 13,000 rpm for 12 min at room temperature and the supernatant liquid transferred to autosampler vials. Metabolites were separated using a Waters Acquity Ultra Performance Liquid Chromatography (UPLC) system (Milford, MA). Sample aliquots of 5 μL rat urine were injected onto a Waters BEH C18 (2.1 mm × 10 cm, 1.7 μm) column held at 40°C. The mobile phase consisted of 0.1% formic acid in water (solvent A) and 0.1% formic acid in acetonitrile (solvent B). The metabolites were eluted using a tri-linear gradient of 0–30% B over 0–6 min, 30–50% B over 6–9 min, and 50–95% B over 9–11 min. The mobile phase was then held constant at 95% B for 1 min before returning to 100% A at 12.1 minutes, after which the system was allowed to equilibrate for 3 minutes before the next sample injection. A constant flow rate of 0.15 mL/min was used throughout the analysis. After each injection, a needle wash cycle consisting of 50:50 acetonitrile/water followed by 0.1% formic acid in water was used to eliminate carry-over and prepare the syringe for the next injection.

### Mass spectrometry

Mass spectrometry using both positive and negative modes of ionization was applied to the UPLC effluent to analyze all classes of molecules. The mass spectrometric data were collected with a Waters LCT Premier single time-of-flight mass spectrometer (TOF-MS) (Milford, MA) equipped with an electrospray ion source. LCT-Premier was operated in W optics mode with 11,000 resolution using dynamic range extension (DRE). The source temperature was set to 120°C with a cone gas flow of 50 L/h, a desolvation temperature of 200°C and a desolvation gas flow of 550 L/h. A capillary voltage of 3.2 kV for positive mode, 2.6 kV for negative mode and cone voltage of 40 V were employed. A scan time of 0.5 s with an inter-scan delay of 0.05 s was used for all the analyses. Leucine eukephalin at a concentration of 250 pg/μL (in 50:50 acetonitrile:water with 0.1% formic acid) was used as a lock-mass in positive mode ([M+H]^+ ^= 556.2771), and 29 ng/μL for negative mode ([M-H]^- ^= 554.2615). The lock spray frequency was 5 s and the lock mass data was averaged over 10 scans for collection. A full scan mode from m/z 50 to 850 from 0–12 min was used for data collection, in both positive and negative mode. Compounds detected by MS were compared with authentic standards for confirmation.

### NMR data analysis

NMR spectra were processed using ACD/Labs 1D NMR Manager (Toronto, Canada). The raw FIDs were zero filled to 131,072 points, multiplied by a 0.3 Hz exponential function and Fourier transformed. The transformed spectra were then phased using the "simple method" and baseline corrected using the "SpAveraging" method with a box half width of 61 points and a noise factor of 3. All spectra were autoreferenced to the TMSP peak at 0.0 ppm. The spectra were overlaid in the processing window and grouped. Dark regions containing the resonances for water, urea, and other NMR solvent peaks were removed prior to integration. The total NMR intensity without water, urea, TMSP and other solvent regions was recorded and the intensity of the TMSP was recorded separately. The intelligent bucketing module was employed for integration with the bucket width set to 0.04 ppm and the looseness set to 50% for bin size optimization. The table of integrals normalized to total spectra intensity was exported for statistical analysis. The table was also renormalized by three different factors to investigate the effects of normalization on the analysis. The renormalization factors included: weight, 1/weight, and concentration of creatinine.

All statistical analyses of NMR data were done using Statistica version 6.0 (Statsoft, Tulsa, OK). Principle component analysis (PCA) based on covariance of the data was applied to the bucketed intensities. Metabolite identification within the individual spectra was accomplished using the Chenomx NMR Suite (Chenomx, Calgary, Canada), which has a database of >250 compounds. The concentrations obtained by Chenomx analysis were first normalized to the determined creatinine concentration and the data between two different studies compared. The concentrations were also normalized by the TMSP peak intensity divided by the total NMR intensity without water, urea, TMSP and solvent regions. This was done to reduce the effects of TMSP volatility and other experimental errors that result in variance in the TMSP peak since the Chenomx NMR Suite uses the TMSP peak area to quantify the concentrations of the metabolites detected in the spectra.

### LC/MS data analysis

Raw data were analyzed using Micromass MarkerLynx Application version 4.0 (Waters Corporation). MarkerLynx was employed for peak finding and peak alignment and reports the mass, retention time (t_r_) and intensity of the peaks. The original data were processed using the following parameters: initial retention time 0 min, final retention time 12 min, mass tolerance 0.02 Da, retention time tolerance 0.1 min and 20 masses in a 0.2 min retention window. The raw data was then transformed into a single matrix containing aligned peaks with the same mass/retention time pair along with peak normalized intensities and sample name. The resulting two-dimensional data were analyzed by PCA.

## List of abbreviations

Crea = Creatinine

DRE = Dynamic range extension

EIC = Extracted ion chromatogram

HPLC/MS = High-performance liquid chromatography/mass spectrometry

LC/MS = Liquid chromatography/mass spectrometry

NMR = Nuclear magnetic resonance

PC = Principal component

PCA = Principal component analysis

ROS = Reactive oxygen species

SD = Sprague-Dawley

TIC = Total ion chromatogram

TMSP = Sodium 3-trimethyl-silyl-[2,2,3,3,-d_4_]propionate

TOF = Time of Flight

t_r _= Retention time

μM = Micromolar

UPLC/MS = Ultra-performance liquid chromatography/mass spectrometry

## Competing interests

The authors declare that they have no competing interests.

## Authors' contributions

LS acquired and analyzed the NMR-based metabolomics data. JS acquired and analyzed the MS-based metabolomics data. PE designed the study and provided samples to NCTR for analysis. RH provided MS validation for significant metabolites found by UPLC/MS. JH and RB coordinated the study. All authors helped to draft, proof, and approve the final manuscript.

## Supplementary Material

Additional file 1 **3D NMR PCA scores plot**. The scores plot for 3D PCA based on covariances of the NMR data from 25-day (red circles), 40-day (blue circles), and 80-day (yellow circles) old SD rats at all time points. Click here for file

Additional file 2 **Representative positive mode MS total ion chromatograms**. Typical positive ion mode total ion chromatograms (TIC) for 25-day old, 40-day old and 80-day old SD rats. The mass spectrum of hippuric acid (*m/z *180.0661, t_R _5.85 min, mass accuracy 0.7 ppm) is displayed as an inset. Click here for file

Additional file 3 **Representative negative mode MS total ion chromatograms**. Typical total ion chromatograms (TIC) for 25-day old, 40-day old, and 80-day old SD rats from UPLC/MS analysis in negative ionization mode. The mass spectrum of hippuric acid (*m/z *178.0500, t_R _5.85 min, mass accuracy 2.8 ppm) is shown as an inset. Click here for file
