# Supplementary figures and images for: Metabonomics evaluations of age-related changes in the urinary compositions of male Sprague Dawley rats and effects of data normalization methods on statistical and quantitative analysis
Source: BMC Bioinformatics. 2007 Nov 1;8(Suppl 7):S3. doi: 10.1186/1471-2105-8-S7-S3 (PMC2099495; doi:10.1186/1471-2105-8-S7-S3)

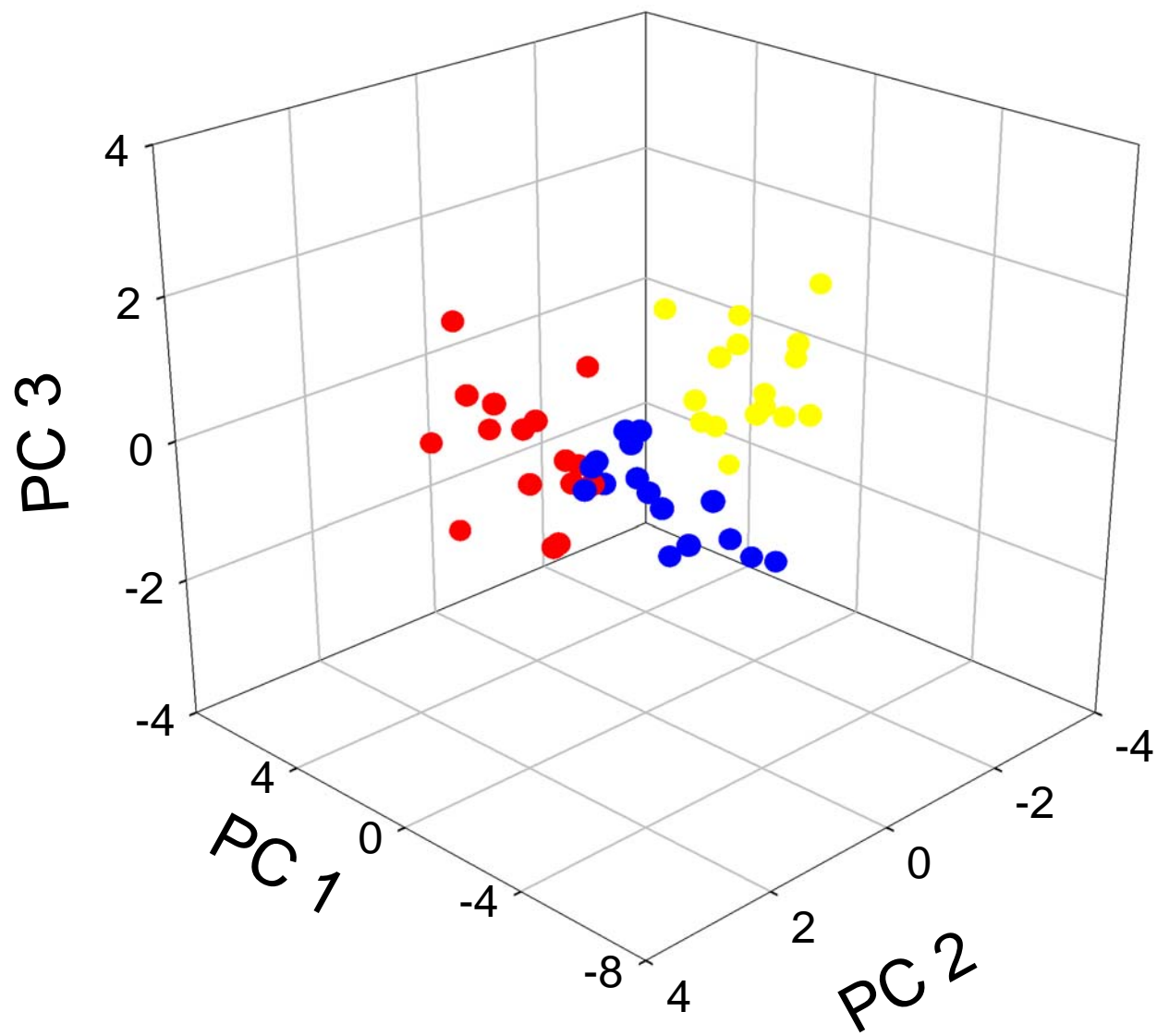

Supplement: Additional file 1 — 3D NMR PCA scores plot. The scores plot for 3D PCA based on covariances of the NMR data from 25-day (red circles), 40-day (blue circles), and 80-day (yellow circles) old SD rats at all time points. [file 1471-2105-8-S7-S3-S1.pdf]

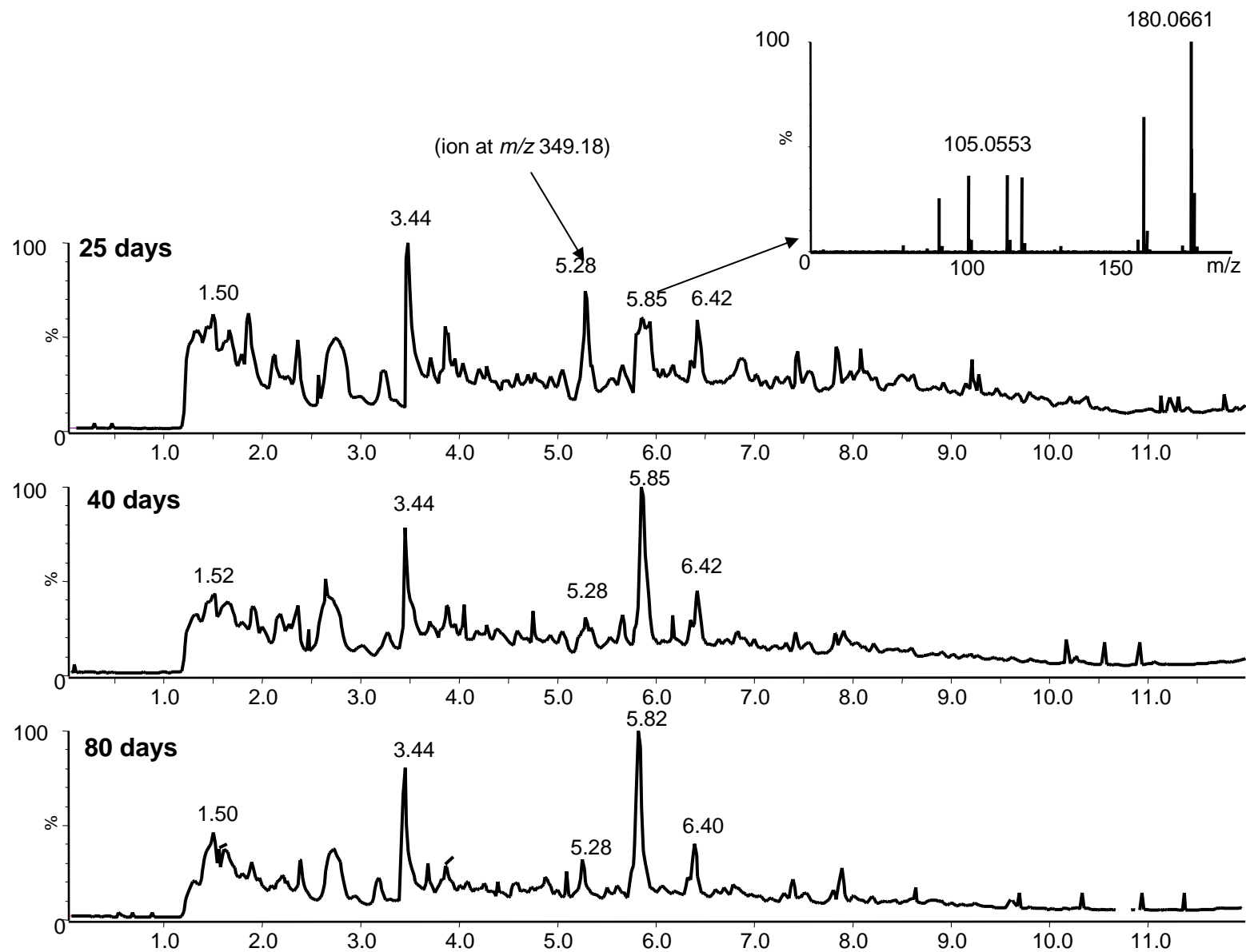

Supplement: Additional file 2 — Representative positive mode MS total ion chromatograms. Typical positive ion mode total ion chromatograms (TIC) for 25-day old, 40-day old and 80-day old SD rats. The mass spectrum of hippuric acid (m/z 180.0661, tR 5.85 min, mass accuracy 0.7 ppm) is displayed as an inset. [file 1471-2105-8-S7-S3-S2.pdf]

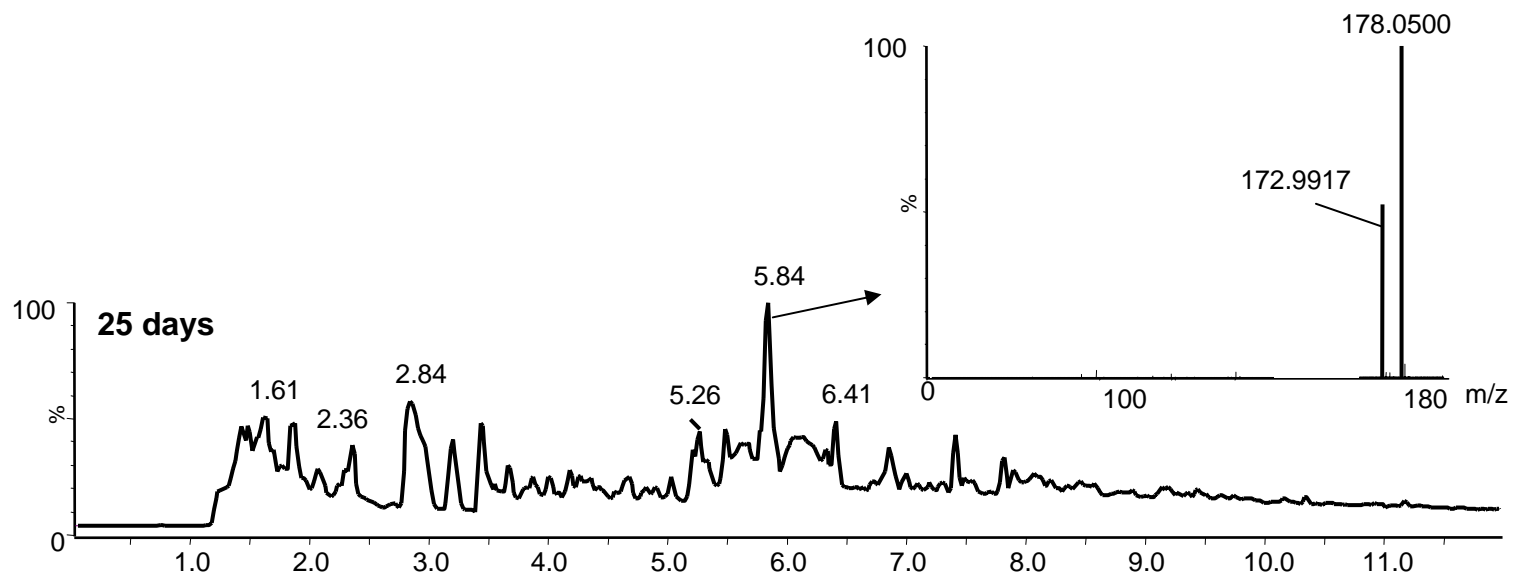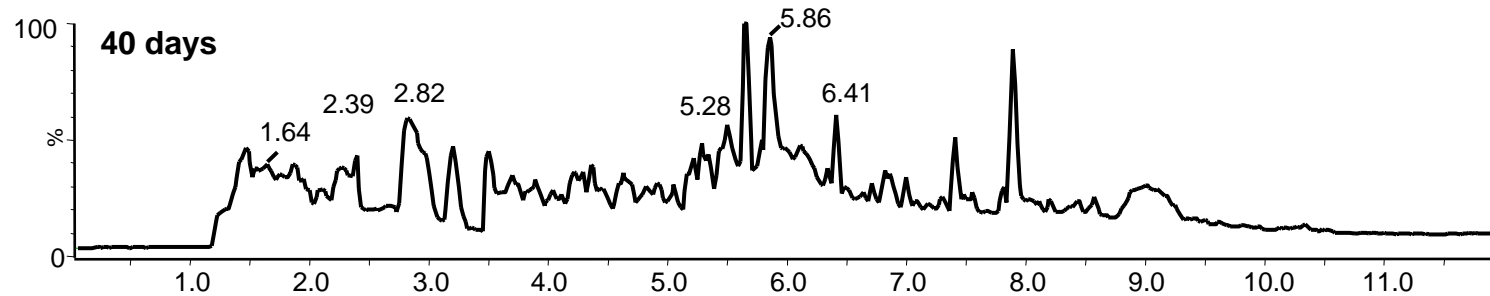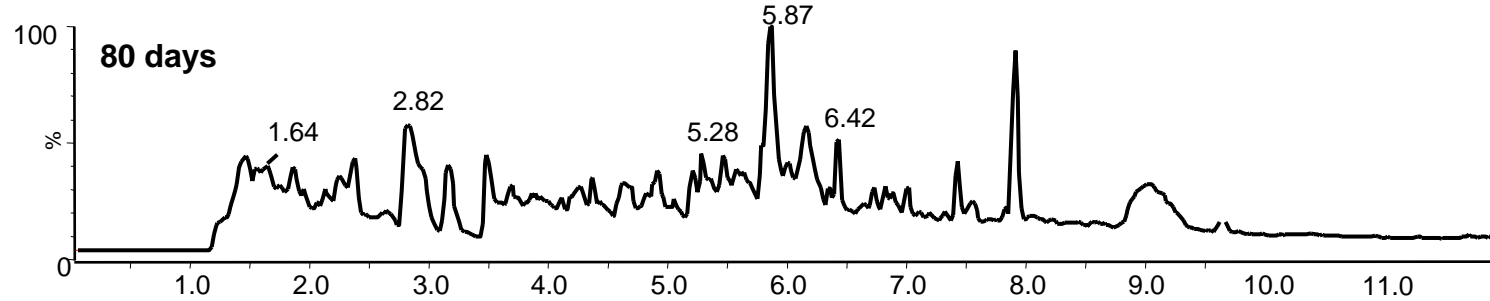

Supplement: Additional file 3 — Representative negative mode MS total ion chromatograms. Typical total ion chromatograms (TIC) for 25-day old, 40-day old, and 80-day old SD rats from UPLC/MS analysis in negative ionization mode. The mass spectrum of hippuric acid (m/z 178.0500, tR 5.85 min, mass accuracy 2.8 ppm) is shown as an inset. [file 1471-2105-8-S7-S3-S3.pdf]
